# Dynamics of Prolyl Hydroxylases Levels During Disease Progression in Experimental Colitis

**DOI:** 10.1007/s10753-019-01065-3

**Published:** 2019-08-03

**Authors:** Hamid A. Bakshi, Vijay Mishra, Saurabh Satija, Meenu Mehta, Faruk L. Hakkim, Prashant Kesharwani, Kamal Dua, Dinesh K. Chellappan, Nitin B. Charbe, Garima Shrivastava, S. Rajeshkumar, Alaa A. Aljabali, Bahaa Al-Trad, Kavita Pabreja, Murtaza M. Tambuwala

**Affiliations:** 1 SAAD Centre for Pharmacy and Diabetes, School of Pharmacy and Pharmaceutical Science, Ulster University, Coleraine, County Londonderry, Northern Ireland BT52 1SA UK; 2 School of Pharmaceutical Sciences, Lovely Professional University, Phagwara, Punjab India; 3 Department of Mathematics and Sciences, College of Arts and Applied Sciences, Dhofar University, Salalah, Oman; 4 School of Pharmaceutical Education and Research, Jamia Hamdard (Hamdard University), New Delhi, 110062 India; 5 Discipline of Pharmacy, Graduate School of Health, University of Technology Sydney, Sydney, NSW 2007 Australia; 6 Department of Life Sciences, School of Pharmacy, International Medical University, Bukit Jalil, 57000 Kuala Lumpur, Malaysia; 7 Departamento de Química Orgánica, Facultad de Química y de Farmacia, Pontificia Universidad Católica de Chile, Av. Vicuña McKenna 4860, 7820436 Macul, Santiago Chile; 8 Sri Adichunchunagiri College of Pharmacy, Sri Adichunchunagiri University, BG Nagar, Karnataka 571418 India; 9 Indian Institute of Technology, Delhi, India; 10 Department of Pharmacology, Saveetha Dental College and Hospitals, SIMATS, Chennai, Tamil Nadu 600077 India; 11 Faculty of Pharmacy, Department of Pharmaceutical Sciences, Yarmouk University, Irbid, Jordan; 12 Department of Biological Sciences, Yarmouk University, Irbid, 21163 Jordan; 13 School of Medicine and Public Health, University of Newcastle, Newcastle, Australia

**Keywords:** prolyl hydroxylases, inflammatory bowel disease, colitis, disease activity index

## Abstract

Hypoxia inducible factor (HIF)-prolyl hydroxylase (PHD) inhibitors are shown to be protective in several models of inflammatory bowel disease (IBD). However, these non-selective inhibitors are known to inhibit all the three isoforms of PHD, *i.e.* PHD-1, PHD-2 and PHD-3. In the present report, we investigated the associated changes in levels of PHDs during the development and recovery of chemically induced colitis in mice. The results indicated that in the experimental model of murine colitis, levels of both, PHD-1 and PHD-2 were found to be increased with the progression of the disease; however, the level of PHD-3 remained the same in group of healthy controls and mice with colitis. Thus, the findings advocated that inhibitors, which inhibited all three isoforms of PHD could not be ideal therapeutics for IBD since PHD-3 is required for normal gut function. Hence, this necessitates the development of new compounds capable of selectively inhibiting PHD-1 and PHD-2 for effective treatment of IBD.

## INTRODUCTION

Hypoxia-inducible factor (HIF)-prolyl hydroxylases (PHDs) are members of the dioxygenase enzymes family. These enzymes are known to play an important function in intracellular oxygen sensing as well as signalling responses in low oxygen levels (hypoxia). This occurs mainly by the regulation of HIF stability [1–4]. These PHDs consist of three isoforms (PHD-1, PHD-2 and PHD-3), which share mostly similar biochemical characteristics; however, all three isoforms of PHDs are known to have very distinct and tissue-specific expression profiles and, regulation of HIF, which in turn can affect pro-survival signalling pathways, including nuclear factor κB pathways during inflammation [1, 5–7]. These PHDs are known to mediate their effect on HIF pathway *via* hydroxylation of proline residues of the HIF*α* subunit, which is degraded under normoxic condition. Once it is hydroxylated, HIF*α* acts as a target for ubiquitylation by von Hipple Lindau E3 ubiquititin ligase and results in the degradation and ubiquitination of HIF*α* [8]. The PHDs perform a pivotal function in an adaptive response known to occur during hypoxia *via* the activation of several pathways of genes expression implicated in support of cell survival, erythropoiesis, angiogenesis and metabolism. These features make PHD an interesting target for therapy [9].

Inflammatory bowel disease (IBD), an idiopathic disease with genetically heterogenous nature, has chronic inflammatory conditions in the gastrointestinal tract (GIT) with severe pathology and limited therapeutic options [10–12]. To date, the underlying causes of IBD are not well-defined; however, the fundamental defect in this disease involves damage/injury of the intestinal epithelial barrier resulting in the development and progression of the disease. Hence, targeting the HIF-PHD pathway may be a fascinating area for potential future therapeutics for inflammatory disease.

Various reports employing pharmacological inhibition of hydroxylase enzyme revealed improvisation in a variety of inflammatory conditions including IBD [13, 15]. Several researchers reported that pan-hydroxylase inhibitors such as dimethyloxalylglycine (DMOG) have a protective effect in experimental colitis [13–15]. However, the majority of these studies have not identified the actual isoform of PHDs or the effector pathways involved in this protective effect.

Our previous findings suggested that there was an increased PHD-1 level in patients with active ulcerative colitis and genetic loss of PHD-1 was found to be protective in experimental colitis [16]. However, since there are three isoforms of PHDs, it would be of interest for researchers to know how the levels of PHDs change during disease development and recovery in IBD. Thus, to answer this query, the present study has been framed to examine the changes in levels of PHDs (PHD-1, -2 and -3) during the inflammation development and its progression in an experimental model of murine colitis.

## MATERIALS AND METHODS

### Dextran Sodium Sulphate (DSS)-Induced Colitis

Colitis was induced in female C57/Bl6 mice using our previously established protocol [15, 17]. Briefly, 2.5 % w/v dextran sodium sulphate (DSS) was administered in mice *via* drinking water to induce colitis. Colon tissues were taken at days 2, 3, 4, 5, 6 and 7 from mice exposed to 2.5 % w/v DSS in drinking water. The mice of the recovery group were exposed to 2.5 % w/v DSS in the drinking water for 5 days and allowed to recover naturally for the next 5 days. On the tenth day, the colon tissue was collected from this group.

### Assessment of Prolyl Hydroxylases (PHD) Levels

The colon tissue lysates were analysed for the level of protein expression of PHD-1, -2 and −-3 levels by a Western blotting technique as reported previously [16].

### Disease Activity Index (DAI)

The weight of each animal was recorded on a daily basis. The stool consistency and appearance of faecal blood was monitored. These parameters were converted into the disease activity index (DAI) as illustrated previously [18].

### Colon Length

Each excised colon length was recorded after removal of the faecal matter using a cotton swab dipped in the phosphate-buffer saline (PBS) solution following the previously reported method [15, 18].

### Statistical Analysis

The analysis of variance (ANOVA) or Student’s *t* test was employed for statistical comparisons and data was represented as mean ± standard deviation (mean ± SD).

## RESULTS AND DISCUSSION

**Fig. 1:**
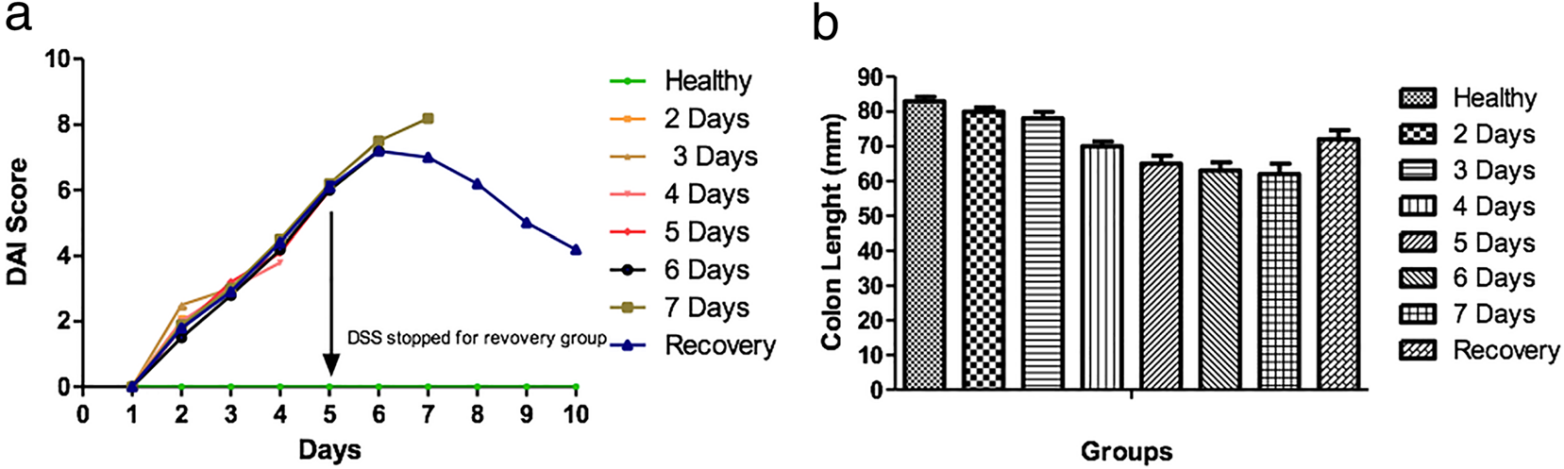
Changes in disease activity score (DAI) and colon length during active colitis. In **a**, the composite score of weight loss, stool consistency and blood in faeces during disease progression was represented, and in **b**, the changes of colon length during active colitis were given. Each control and experimental group contained a minimum of *n* = 6 mice.

The DAI scores indicated that administration of DSS in drinking water caused weight loss, diarrhoea and appearance of blood in faeces, since the DAI steadily raised from day 2 onwards indicating the onset of inflammation from day 2 (Fig. 1a). We have also noted that the colon length of mice progressively decreased as the inflammation progressed (Fig. 1b). This could be due to muscle wasting of the colon tissue during inflammation. Furthermore, both the DAI score and colon length indicated that withdrawal of DSS from the drinking water resulted in tissue recovery and alleviation of the classical symptoms of colitis (Fig. 1).

**Fig. 2:**
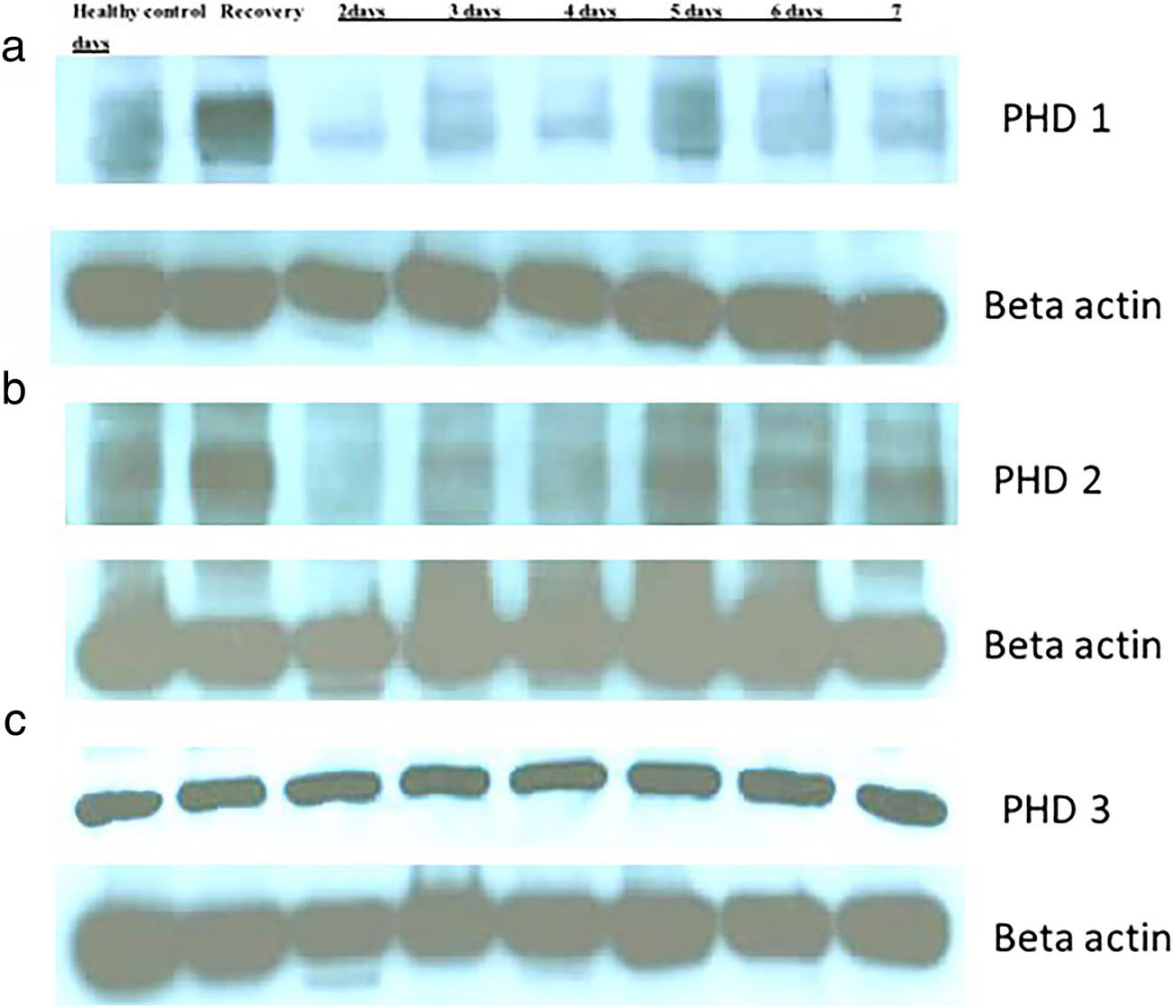
Colon homogenates assessed for **a** PHD-1, **b** PHD-2 and **c** PHD-3 protein levels. Each control and experimental group contained a minimum of *n* = 6 mice. PHD-1 level was found to be increased with disease progression in murine model of colitis.

Our key results indicated that PHD-1 level was found to be lower than that of the healthy control at day 2 and it gradually increased by day 5 (Fig. 2). This event suggested that PHD-1 expression levels initially decreased when inflammation sets in and then progressively increased as the inflammation was established due to the administration of DSS in the drinking water. The recovery group has shown the highest level of PHD-1 expression in the colon tissue (Fig. 2a), suggesting that once the PHD-1 level is elevated due to inflammation, it does not go down even after the withdrawal of DSS. The PHD-2 levels followed a similar decline trend at day 2 followed by an increase at day 5. In the case of PHD-2, the recovery group also showed the highest levels in the colonic tissue (Fig. 2b). Contrary to this, the levels of PHD-3 were observed to be constant in healthy control, recovery group and during the course of disease progression (Fig. 2c). It suggested that PHD-3 has not involved in the development and progression of inflammation during experimental colitis.

The upregulated PHD-1 in colonic biopsies of IBD patients illustrated the correlation between PHD1 expression and disease severity in patients [16, 19]. Among PHD-1, -2 and -3, only PHD-1 deficient mice were found to be selectively protected against DSS-induced colitis development advocating an affirmative function of PHD-1 in the management of intestinal epithelial cell apoptosis and preservation of epithelial barrier during intestinal inflammation [16]. The hypothesised link between improved barrier function and consequent protection against the colitis development was also supported by studies performed in other models, indicating an antiapoptotic effect of pharmacologically inhibited hydroxylase enzyme [20–22].

## CONCLUSION

These results suggest that PHD-3 isoform is expressed in healthy as well as the diseased colon tissue, indicating that PHD-3 is required for the normal function of the gut. However, PHD-1 and -2 expression levels fall when inflammation is initiated and gradually increases during the progression of inflammation and is observed to be the highest in chronic stages. The recovery group indicates that once the agent causing inflammation of the colon, *i.e.* DSS is removed from the drinking water, these mice show signs of recovery (Fig. 1); however, the increased levels of PHD-1 and -2 are not reduced to normal level, which suggest that PHD-1 and -2 are involved in chronic inflammatory stages of the disease. These findings advocate that inhibition of all PHDs using a non-selective prolyl hydroxylases inhibitor may not be the best strategy towards the development of new therapeutics for IBD. Hence, strategies to selectively target PHD-1 and -2 are warranted leading to an improved therapeutic advancement in the field of IBD treatment.
